# Applying the Consolidated Framework for Implementation Research (CFIR) to understand college health administrator perceptions on adopting and implementing opioid overdose education and naloxone distribution (OEND) programs among universities nationally

**DOI:** 10.21203/rs.3.rs-7313339/v1

**Published:** 2025-08-21

**Authors:** Savannah P. Alexander, Elizabeth Shelton, Matthew Lee, G Tharp, Michael McNeil, Melanie Bernitz, Kevin Graves, Lisa R. Metsch, Rachel C. Shelton

**Affiliations:** Columbia University Mailman School of Public Health; Columbia University Irving Medical Center; New York University (NYU) Grossman School of Medicine/NYU Langone Health; Vynamic, LLC: Health Industry Management Consulting; Columbia University; Columbia University Vagelos College of Physicians and Surgeons; Kevin Graves Marriage and Family Therapist Inc; Columbia University; Columbia University Mailman School of Public Health

**Keywords:** Opioid overdose education, naloxone distribution, opioid, naloxone, harm reduction, implementation science, higher education

## Abstract

**Background:**

The United States opioid epidemic’s reach is expanding. Rapidly scaling opioid education and naloxone distribution (OEND) programs is essential within a multipronged public health response. Universities offer infrastructure with potential to support routine, widespread OEND implementation among adolescents and young adults nationally, a priority population who could disseminate to broader networks and geographic communities. This important setting is underutilized, and critical gaps remain in understanding university-based OEND program adoption/implementation.

**Methods:**

We conducted semi-structured, in-depth interviews (n=21) among a purposively selected national sample of college health administrators to understand their perceptions of barriers/facilitators of implementing OEND programs at their universities and among universities nationally. The Consolidated Framework for Implementation Research guided data collection and inductive-deductive thematic analysis.

**Results:**

Regarding *Relative Priority* (*Inner Setting*), *High/Mid-Level Leaders (Individuals)*, and *Rationale for the Intervention* (emergent code in the *Innovation* domain), participants described the need for compelling justification to adopt, implement, and prioritize university-based OEND programs. The key justification for administration was student opioid overdose and mortality events. Absent these events and regarding *Assessing Needs* (*Implementation Process*), participants described the need for student opioid misuse data to justify investment. Regarding *Local Conditions* (*Outer Setting*) and *Tension for Change* (*Inner Setting*), participants indicated that a university’s level of community obligation and integration determined which opioid overdoses and misuse data administration deemed relevant to justify adoption. Regarding *Partnerships & Connections* (*Outer Setting*), *Relational Connections* (*Inner Setting*); *Planning*, *Engaging*, and *Teaming* (*Implementation Process*), participants described external/internal collaborators’ key roles in OEND program adoption/implementation. Regarding *Local Attitudes* (*Outer Setting*), participants described the need to manage political risk, implicating stigma against harm reduction programming. Regarding *Culture* and *Available Resources* (*Inner Setting*), participants illustrated a trajectory in which their institutions prioritized recovery programming for years before prioritizing harm reduction programming (e.g., OEND programs).

**Conclusions:**

Our findings underscore the complexity of university-based OEND program implementation while providing actionable insights to support its national scale-up. Building on identified distinctions between non-implementing and implementing universities, future research should establish OEND programming implementation phase among universities nationally, advance understanding of implementation determinants and strategies distinguishing each phase, and establish core components and best practices.

## Background

The United States opioid overdose epidemic is evolving as fentanyl permeates the illicit drug supply, increasing the epidemic’s geographic and sociodemographic reach compared to its earlier waves (e.g., due to intentional and unintentional exposure among people who use drugs [PWUD] and unintentional, incidental exposure among others) (1–5). Fentanyl’s potency (e.g., ~ 50 times greater than heroin), pharmacodynamics (e.g., rapid crossing of the blood–brain barrier), and severe physiological impact (e.g., near immediate respiratory suppression) render it highly lethal (6), and its lethality is exacerbated by rising fentanyl-involved polysubstance use (e.g., with cocaine, xylazine) (7–10). Between 2019 and 2022, years of life lost due to opioid overdose deaths increased from approximately 2.0 to 3.1 million (38 years lost per death), with years lost per capita increasing in most states and more than doubling in 16 states (11). Despite notable decreases in drug overdose deaths beginning in 2023, overdose remains the leading cause of death among Americans aged 18–44 (12). Further, recent data suggest drug overdose deaths are again increasing, with synthetic opioids being the primary drug involved (13, 14).

The lethality of rising fentanyl-involved polysubstance use and unprecedented reach of the current opioid epidemic wave renders rapid expansion of evidence-based harm reduction services essential as part of a multipronged public health response (e.g., including expansion of evidence-based recovery services (15–19)). Opioid education and naloxone distribution (OEND) programs are a key harm reduction service, providing training to laypersons to respond to opioid overdoses and administer naloxone (opioid antagonist that reverses opioid-induced respiratory suppression) (20–22). A 2025 systematic review and meta-analysis found that community-based OEND programs effectively reduce opioid overdose deaths at individual, city, county, and state levels (23). However, OEND program scale is inadequate, as naloxone saturation modeling studies demonstrate insufficient naloxone distribution nationally (a key OEND program outcome) (24–26).

In OEND program scaling up efforts, adolescents and young adults (AYAs) must be prioritized as **1)** previously rising opioid overdose and mortality in this age group was exacerbated by the COVID-19 pandemic (27–31), **2)** laws/policies supporting naloxone access alone have not effectively reduced that mortality (32), **3)** AYAs have limited access to medications/treatment for opioid use disorder, putting them at increased overdose risk (33, 34), **4)** AYAs are at considerable risk of unintentional, incidental fentanyl exposure (e.g., experimentation with illicit drugs that are increasingly combined with fentanyl) (35), and **5)** AYAs have very limited knowledge of naloxone (36). Based on nationally representative data, fentanyl-involved mortality among those aged 20 and younger was 30 times greater in 2021 than in 2013 (37). Among those aged 10–19 who died due to drug overdose (primarily fentanyl-related) between July-December 2019 and July-December 2021, the majority did not have an opioid use history, and most bystanders (present in 2/3 of the drug overdose deaths) did not intervene (38). Further, based on a national study among 18–25 year-olds attending higher-education institutions, less than 15% know how to administer naloxone (36).

As the sustainability of national efforts (e.g., State Opioid Response Grants) is uncertain amidst federal funding changes (39), colleges and universities (hereafter referred to as ‘universities’) offer existing infrastructure with potential to support OEND program implementation among AYAs nationally (40). Training AYAs will facilitate enhanced response to opioid overdoses and administration of naloxone amongst themselves and their broader social networks (e.g., considering social networks’ strong influence among AYAs; (41–44)), thereby supporting the widespread geographic reach and impact of university-based OEND programs (40). While some universities began implementing OEND programs several years ago (e.g., (45)), this setting remains critically underutilized. There is a lack of theoretically-informed implementation research on university-based OEND programs (46) and limited application of implementation science theories and frameworks in similar settings (e.g., community-based OEND programs, university-based substance use programs) (47–52). Research is needed to better understand implementation determinants of university-based OEND programs to support their national scale-up. As such, informed by The Consolidated Framework for Implementation Research (CFIR) (53–55), we conducted and analyzed interviews among a national sample of college health administrators to understand their perceptions of the barriers and facilitators of implementing OEND programs at their universities and among universities nationally.

## Methods

### Data Collection

**Figure 1:**
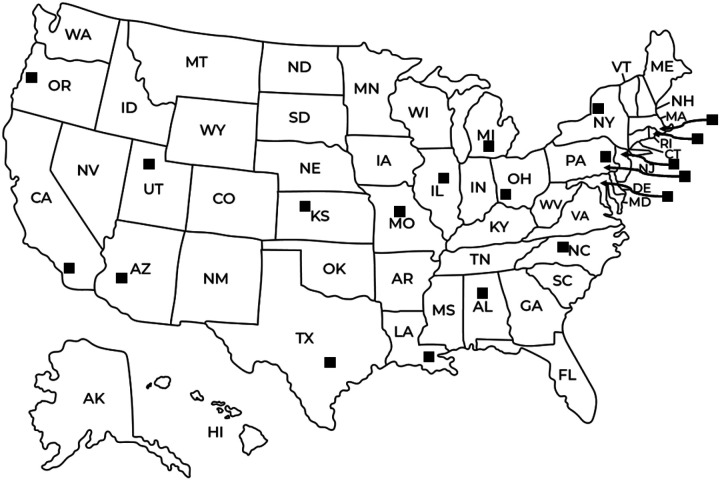
Geographical distribution of represented universities and substance use consortium (n=20)

Between June and November 2020, trained qualitative researchers (ES, GT, ML) conducted in-depth interviews among purposively selected college health administrators (n = 21) from 19 universities nationally and a statewide higher education substance use consortium (24 member universities), as they have relevant expertise and decision-making power with respect to university-based OEND programming. We used purposive selection criteria at the university and college health administrator level to ensure data collection within a sample similar enough to yield consistent thematic findings and diverse enough to understand the range of facilitators and barriers to university-based OEND program implementation and scale-up. We describe all purposive selection criteria and corresponding university/administrator characteristics in Table 1. Figure 1 visualizes the geographic distribution criterion. Participants were recruited through 3 rounds of individual emails disseminated among American College Health Association (ACHA) and Student Affairs Administrators in Higher Education (NASPA) member networks by a researcher who is an ACHA and NASPA member and Program Director of Columbia University’s OEND program (MM).

We applied CFIR as it is an evidence-based implementation science determinants framework with demonstrated ability to facilitate comprehensive, multilevel assessments of implementation determinants needed to inform actionable implementation strategies (53–55). We operationalized the 5 overarching domains as follows: 1) *Outer Setting* (multi-level context including communities, regions, states, and nation surrounding the universities), 2) *Inner Setting* (university campuses), 3) *Intervention Characteristics* (university-based OEND programs), 4) *Characteristics of Individuals* (broadly including individuals in the Outer and Inner Settings with decision-making influence over or potentially impacted by university-based OEND program implementation), and 5) *Process* (processes facilitating university-based OEND program implementation). CFIR 1.0, the version published at the time of data collection (53), informed the interview guide (Additional File 1 maps questions/probes onto CFIR 1.0). Interviews were audio-recorded, transcribed verbatim, and lasted 22–64 minutes (average: 41 minutes). We adhered to the Consolidated Criteria for Reporting Qualitative Research (COREQ) checklist (Additional File 2).

### Data Analysis

**Figure 2:**
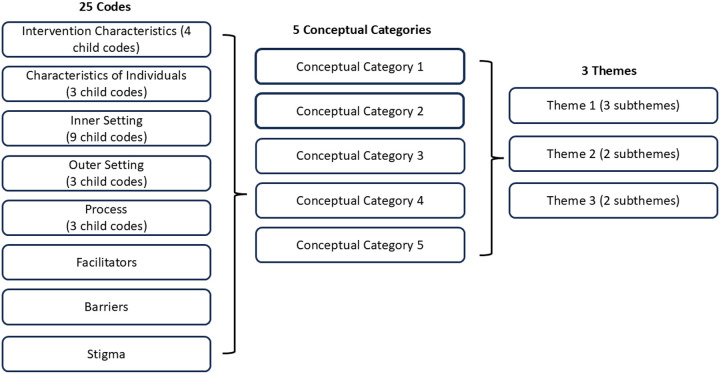
Combined inductive-deductive qualitative data analysis process

CFIR 1.0 informed initial analyses. We used an inductive-deductive approach (56). We included all CFIR 1.0 domains as parent codes, applicable CFIR 1.0 constructs as child codes, and other parent/child codes identified through coding but that did not map onto CFIR 1.0. The codebook was imported into NVivo v12; two researchers (SPA, ES) coded all interviews in parallel. SPA and ES iteratively refined the codebook and adjusted prior coding as needed, with no codebook adjustments after the eleventh transcript. SPA and ES conducted regular coding reviews and resolved discrepancies through discussion and consensus, informed by two triangulation meetings with implementation science experts (RCS, ML). SPA and ES grouped codes into five salient conceptual categories and iteratively reviewed all data coded at each category as well as their respective memos to synthesize themes/subthemes. SPA and ES finalized themes/subthemes after discussion with an implementation science expert (RCS) and additional review of their memos organized by conceptual category, referencing transcripts as needed. Figure 2 summarizes this process (57).

The formulation of conceptual categories elucidated relationships between emergent codes and existing CFIR 1.0 constructs, demonstrating meaningful intersections across CFIR 1.0 domains. During theme/subtheme formulation, CFIR 2.0 was published (54), and upon additional analysis, we determined that several emergent codes that had not mapped onto CFIR 1.0 mapped onto CFIR 2.0 (see Additional File 3 for details). Thus, we present our final themes/subthemes below in relation to CFIR 2.0.

We investigated potential differences based on our purposive selection criteria (Table 1; e.g., private versus public universities), which did not yield meaningful findings. However, we determined key thematic patterns differed according to universities’ respective OEND program implementation phase, which CFIR does not directly consider. Specifically, we found meaningful differences between two implementation phases, broadly informed by the Exploration, Preparation, Implementation, Sustainability (EPIS) Framework (58, 59): **universities without plans to implement an OEND program** (had not explored the possibility of implementation (n = 4), were exploring that possibility (n = 2), had decided against implementation (n = 4)), and **universities in the process of OEND program implementation** (actively preparing (n = 3), implementing (n = 4), or sustaining it (n = 3)). We refer to the former as “non-implementing universities” and the latter as “implementing universities” hereafter.

## Results

Below we describe the identified themes/subthemes, demonstrating meaningful intersections across CFIR 2.0 domains and constructs. We explain key differences and similarities within each theme/subtheme by implementation phase and describe salient divergent examples. Table 2 summarizes these key findings and provides additional illustrative quotes. Of note, though study participants are administrators, they discussed administration as an overarching university entity with decision-making power (e.g., including Deans). As such, “administration” refers to this overarching entity at universities, and when relevant, we refer to “participants” specifically.

### Theme 1: Justification that Compels Administration to Act: Reactive Orientation and University Purview

Related to *Relative Priority* (*Inner Setting* domain), *High/Mid-Level Leaders (Individuals* domain*)*, and *Rationale for the Intervention* (emergent code in the *Innovation* domain), participants described the need for compelling justification to adopt, implement, and prioritize university-based OEND programs. The key justification for administration was student opioid overdose and mortality events. Absent these events and related to *Assessing Needs* (*Implementation Process* domain), participants described the need for student opioid misuse data to justify investment. Related to *Local Conditions* (*Outer Setting* domain) and *Tension for Change* (*Inner Setting* domain), participants indicated that a university’s level of community obligation and integration determined which opioid overdoses and opioid misuse data (e.g., student, community, on/off campus) the administration deemed relevant to justify adoption. These subthemes are described in detail below.

#### Subtheme A: Reaction to Student Opioid Overdose and Mortality Events

##### Implementing Universities.

Among implementing universities, student opioid overdose deaths constituted a strong impetus to swiftly implement or more strongly prioritize OEND programs. As an important example from a university with a particularly robust OEND program (e.g., in terms of longevity, number of internal/external partners, ongoing program evaluation and adaptation, expanding recruitment efforts), the administration had previously agreed to educate resident advisors (RAs) on opioid overdoses but did not allow them to carry naloxone. Subsequently, a local community harm reduction group raised awareness about student opioid overdose deaths about which the administration had been unaware. The administration promptly agreed to train RAs on and equip them with naloxone, and over time, allowed the OEND program to grow to include all interested students, staff, and faculty. Another key example illustrates that even in the event of student opioid overdoses, incremental implementation, with naloxone training/distribution as a last step, might be necessary. The administration at one of the implementing universities that experienced student opioid overdoses and deaths agreed to swiftly implement campus-wide opioid and naloxone education among students. Only after several years of the campus substance use committee championing that program (e.g., discussing community overdose and fentanyl data with campus stakeholders) and the hiring of a new university Vice President with relevant clinical background, did the administration agree to implement naloxone training/distribution among students beyond the student ambulance service.

##### Non-Implementing Universities.

In contrast, multiple participants from non-implementing universities explained that administration did not implement an OEND program despite student opioid overdose events. Participants cited low opioid misuse prevalence, low number of opioid overdoses, and lack of opioid overdose death among students, off-campus student overdoses being beyond university purview, and the adequacy of campus or nearby first responders to respond to infrequent student opioid overdoses. Relatedly, participants cited greater relevance of OEND programs to surrounding communities where more opioid overdoses were occurring and into which students were not integrated, and the lack of student outcry about the need for an OEND program. Juxtaposed to this low perceived need for OEND programs, participants described strong competing priorities (namely, student suicidality and deaths), limited campus resources, the need for internal partnerships to institutionalize an OEND program, liability concerns, and stigma-related optics concerns (implicating stigma against harm reduction programming). Participants also explained their universities’ tendency to focus on mental health programming to the neglect of substance use programming, such as an OEND program, even in response to student opioid overdoses.

#### Subtheme B: Student Opioid Misuse Data

##### Non-Implementing Universities.

Participants from non-implementing universities underscored the importance of sufficient student opioid misuse prevalence data (among their students specifically) to justify OEND program implementation. Some indicated there was insufficient prevalence based on available data, while others acknowledged the need to further investigate multiple data sources (e.g., American College Health Association-National College Health Assessment (ACHA-NCHA) survey data from their campus, data from campus colleagues, local emergency department data involving their students). To underscore this emphasis on compelling data, even after student opioid overdose events, some participants described insufficient student opioid misuse as a barrier to adoption.

However, there were cases among non-implementing universities in which representative data or anecdotal knowledge of rising or ongoing student opioid misuse were insufficient to motivate administration to adopt an OEND program. For example, there was a disconnect between a participant and her university’s Collegiate Recovery Community leader, a naloxone trainer who was “very concerned about opioid use in her community” and “[trying] to train as many people as she [could].” Nonetheless, the participant declined the county health department’s offer to fund and implement an OEND program, explaining that adoption would be more likely “if there was a way to know who needed it.”

##### Implementing Universities.

As a caveat to non-implementing universities’ emphasis on data, participants from implementing universities underscored the limitations of available data (e.g., selection bias due to social desirability or opioid misuse-related dropout). They described the need to identify and reach student subgroups experiencing high opioid misuse or at risk of incidental exposure (e.g., fentanyl lacing) even if opioid misuse among the general student populace was low (e.g., by disaggregating and triangulating data, including qualitative data and anecdotal knowledge of at-risk subgroups through their involvement in campus substance use programs).

#### Subtheme C: Relevant Opioid Overdoses and Opioid Misuse

##### Implementing Universities.

Participants described stronger community obligation and integration among implementing universities, which led them to have a broader purview of responsibility to justify adoption. They underscored the relevance of community opioid misuse/overdose to student opioid misuse/overdose (e.g., given that students spent time in the community and community members spent time on campus). They described the opportunity for students to administer naloxone among themselves and members of the surrounding community (whether on or off campus). As examples of the relevance of the surrounding community: Prior to adoption/implementation, one of the implementing universities experienced unexpected student opioid overdose deaths after a sudden influx of fentanyl in the surrounding community of which the participant had been unaware. She urged universities to join local community task forces so that administration could be promptly notified of changes in the surrounding substance use environment. Another explained that their off-campus, community-based OEND program was part of the university’s work on a local community coalition responding to prevalent community opioid overdoses. The administration explained that improving community health would improve student health, since students were integrated within the community and could access the OEND program there. They were also planning to expand these efforts with on-campus implementation. Another participant explained that their university president had served on a statewide opioid task force and strongly prioritized their OEND program, because he believed the university, as a state institution, was obligated to respond to prevalent opioid overdoses in the surrounding community.

##### Non-Implementing Universities.

Participants from non-implementing universities discussed high opioid misuse or overdose prevalence in the surrounding community, local and statewide opioid/substance use coalitions of which the university was a member, a desire to better integrate with the surrounding community, and off-campus student opioid overdose events. However, in contrast to implementing universities, they did not discuss the relevance of community opioid misuse/overdose to potential student opioid misuse/overdose, did not acknowledge the possibility of students administering naloxone in the surrounding community, or understood off-campus applicability, even among students, as beyond university responsibility. As one participant from a non-implementing university (which has experienced off-campus student opioid overdoses and increases in student and surrounding community opioid misuse) explained,

Unfortunately, there are a lot of students that live off of campus…but then that falls out of the purview many times of…the university’s responsibility…But getting the students trained may, would benefit there as well—although they may not have the naloxone themselves.

##### Divergent Examples of Proactive Approaches.

Only two participants, both from implementing universities, conceptualized OEND programs as a widely applicable public health measure to prevent, rather than respond to, student or community opioid overdose and mortality. One explained the need for universities to be aware of and respond to opioid misuse among local residents to build relationships with those communities. The other conceptualized off-campus applicability of their OEND program even more broadly,

People travel from all over the country to attend here and the knowledge goes with them. So, if they’re on a bus, or a train, or plane on their way back to Iowa or California and something happens, they can act.

### Theme 2: External and Internal Partnerships are Needed to Support Adoption and Implementation

**Figure 3:**
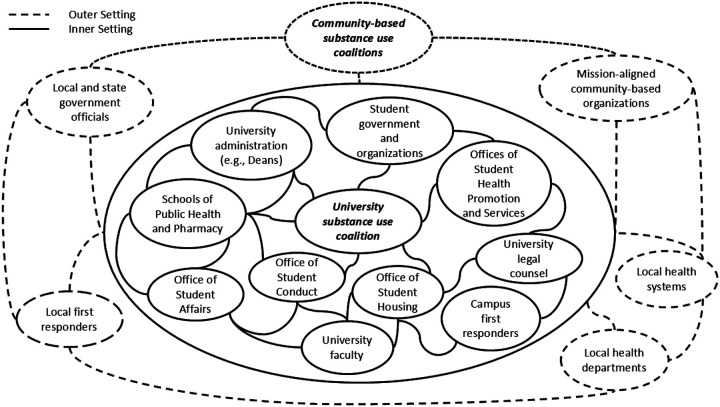
Collaborations among university, community, local, and state individuals and organizations to support OEND program implementation* *This figure is intended to illustrate the myriad internal and external partnerships/collaborations (with the university campus being operationalized as the inner setting) that participants discussed across the interviews (Theme 2), which were integral in navigating university administration’s hesitancy/opposition to implementing OEND programming (Theme 1) as well as internal and external cultural implementation barriers (Theme 3). This figure is not intended to be an exhaustive representation of all possible collaborations between university, community, local, and state individuals and organizations.

Participants explained the complexity of adopting and implementing university-based OEND programs, necessitating external and internal partner involvement (Fig. 3).

#### Subtheme A: External Partnerships and Connections

##### Implementing Universities.

Related to *Partnerships & Connections* (*Outer Setting* domain), and *Planning*, *Engaging*, and *Teaming* (three constructs in the *Implementation Process* domain), participants described critical reasons why external partners/collaborators (e.g., local health departments/systems, substance use/harm reduction coalitions, community-based organizations, government, first responders) are critical to engage in OEND program adoption and implementation efforts. Participants, primarily among implementing universities, perceived the following as key functions of these collaborations: **1**) overcoming administrative active pushback or passivity; **2**) allowing students to access an OEND program elsewhere during the university’s Exploration or Preparation, potentially leading to a sustained partnership; **3**) strategically incorporating naloxone training and kits that are readily accessible in the surrounding community; **4**) overcoming funding/resource constraints (e.g., staff, naloxone); **5**) promoting sustainability; **6**) building academic-community relationships; **7**) staying informed on and responding to opioid misuse/overdoses in the surrounding community and among students; **8**) lowering barriers for students who use illicit drugs and are reticent to identify themselves in university settings. Universities sometimes actively partnered with external entities to implement OEND programs (e.g., partnering with the local health department to offer an OEND program in off-campus, community-based locations that were accessible to students). Other times, universities connected with external entities to implement OEND programs but did not create formal partnerships with them (e.g., encouraging students to pick up free naloxone at off-campus pharmacies).

##### Non-Implementing Universities.

This subtheme was primarily discussed by participants from implementing universities, but participants from non-implementing universities described the importance of external partners/collaborators for functions 1, 2, 4, and 7 from the above list for implementing universities: **1**) overcoming administrative active pushback or passivity; **2**) allowing students to access an OEND program elsewhere during the university’s Exploration; **4**) overcoming funding/resource constraints; **7**) staying informed on and responding to opioid misuse/overdoses in the surrounding community and among students. Importantly, as illustrated by the above example of a participant declining the county health department’s offer to lead an OEND program, the willingness of an external partner to implement might be insufficient to facilitate adoption if university administration lacks a compelling justification to do so.

##### Divergent Example of a Spearheading External Partner.

One participant was a statewide higher education substance use consortium leader rather than an administrator of a single university like the other participants. Her experiences illustrate that an external partner might need to lead adoption and implementation. Administrations across the member universities did not see substance misuse as a key determinant of student retention (a view she perceived as common within the surrounding United States region), and as such, dedicated few internal resources to substance use programming. The consortium collected student substance use data, identified the universities with the highest student opioid misuse prevalence, and even when presented with that data, those universities did not devote internal resources to OEND programs (at least in part due to resource constraints). The state consortium strategically used its limited resources by consistently offering programming (e.g., biweekly site visits) at the universities with the highest student opioid misuse prevalence and more limited programming across the member universities (e.g., one-time train-the-trainer naloxone training).

#### Subtheme B: Internal Partnerships and Connections

Related to *Relational Connections* (*Inner Setting* domain) and *Planning*, *Engaging*, and *Teaming* (three constructs in the *Implementation Process* domain), participants from non-implementing and implementing universities described the need for a collaborative of internal partners to adopt and implement OEND programs (e.g., offices of student health promotion/services, affairs, conduct, housing; campus faculty and first responders; student government/organizations; schools of public health and pharmacy; legal counsel).

##### Non-Implementing Universities.

Participants from non-implementing universities emphasized the need for internal partners to gather data and share perspectives to establish the need for an OEND program that would compel administration to adopt it.

##### Implementing Universities.

Those from implementing universities described internal partners (namely, substance use committees) facilitating OEND program planning and implementation (e.g., discussing implementation determinants and strategies) and sustainability (e.g. championing the program, implementing new engagement strategies, creating social work student internships to support implementation, conducting program evaluation).

##### Student Engagement.

Participants suggested student engagement might be critical to this collaborative effort. Several from non-implementing universities underscored their administration’s student responsiveness. For example, one participant explained, “we’re very student responsive”, so, “anecdotal reports through student government, student organizations, any outcry from the students that they need [an OEND program] or want it” would improve likelihood of adoption. Additionally, students had an integral role in adoption at two implementing universities. At one university, a student and the public health association affiliated with public health majors condemned the university in a student newspaper and social media campaign regarding its lack of harm reduction programming and called on the participant’s office to lead implementation. At the other university, the campus chapter of Students for Sensible Drug Policy participated in a campus substance use committee that spearheaded adoption and implementation, prompted student government to pass a resolution to create a standing naloxone order in the campus pharmacy (which the administration honored), and continued to participate in the committee and promote the OEND program.

### Theme 3: Navigating External and Internal Culture

Participants explained the need to navigate external (within the surrounding community, state, region) and internal (within the university) cultural barriers, implicating stigma against harm reduction programming and PWUD populations.

#### Subtheme A: Navigating External Politics and Culture

##### Non-Implementing Universities.

Related to *Local Attitudes* (*Outer Setting* domain), participants from non-implementing universities explained that political risk management concerns (e.g., optics related to regional/state culture, state legislatures/representatives, funders) could prevent adoption, even when administration is aware of ongoing student opioid misuse. For example, one participant explained that student opioid misuse, though uncommon, usually involved polysubstance use and was “more severe” than other forms of substance misuse. Nonetheless, the administration would not adopt an OEND program, primarily due to concerns about the university’s reputation with conservative state representatives in the context of regional stigma (e.g. construing OEND programs as promoting drug use).

##### Implementing Universities.

Among implementing universities, participants indicated that while political risk management was important, it was feasible to handle (e.g., by framing OEND programs as widely applicable public health prevention rather than incrimination of an on-campus problem; by gradually increasing campus and community visibility of the OEND program). For example, the statewide substance use consortium leader explained she had to be careful not to alienate the member universities, while attempting to counter-educate against the state/regional stigma around opioid misuse (e.g., opioid misuse as an individual failing).

#### Subtheme B: Navigating Internal Culture

##### Implementing Universities.

Related to *Culture* and *Available Resources* (*Inner Setting* domain), participants from implementing universities illustrated a trajectory in which their institutions prioritized recovery programming for years before prioritizing harm reduction programming. For example, one participant described her university’s substance free housing program as one of the first in the country and acting as “an extraordinary beacon for people who are in recovery to know that they have a safe harbor.” That university was preparing to implement an OEND program as “an expansion of what [they were] already doing”, though the administration had decided against doing so several years prior. Additionally, the university with a particularly robust OEND program (described above) implemented one of the first Collegiate Recovery Communities in the nation, which they increasingly prioritized over time through diverse on-campus partnerships and institutional funding.

##### Non-Implementing Universities.

This recovery-harm reduction trajectory emerged among two non-implementing universities that had instituted substantial recovery programming and were beginning to institute harm reduction programming, with participants describing the laborious processes required (e.g., five-year effort in collaboration with campus police and state legislature to institute campus Good Samaritan policy). However, other non-implementing universities were not at the point of prioritizing recovery or other substance use programming (e.g., due to a tendency to prioritize mental health over substance use programming, conceptualizing them as separate rather than complementary services; the “normalization” of substance use within the university acting as a strong barrier to substance use programming adoption), which participants perceived as a substantial barrier to OEND program adoption. An important example from an implementing university revealed the danger of such internal barriers, in that the “normalization” of illicit drug use and related student mistrust and disconnect from the university persisted until multiple, unexpected opioid overdose deaths occurred.

## Discussion

### Overarching Findings and Theoretical Advancements

Our findings underscore the complexity of university-based OEND program implementation while providing insight into actionable facilitators to support the national scale-up of this essential evidence-based harm reduction service, namely regarding the key roles of internal and external partners/collaborators. Drawing on all identified themes/subthemes, these roles include **a)** navigating university administration’s hesitancy/opposition to implement OEND programming, **b)** collecting new and compiling existing data to understand the campus substance use environment, **c)** sharing that data and anecdotal evidence with university administration and external stakeholders (e.g., local law/policymakers) to establish program need despite the common perception of low student opioid misuse, **d)** overcoming internal and external cultural barriers implicating persistent stigma against and legal complexity of harm reduction programming, and **e)** overcoming funding and other resource constraints.

These varied roles elucidate relationships between CFIR domains and constructs, as operationalized within the context of university-based OEND program implementation (Table 2, Additional File 3) and provide insights into differences in determinants by implementation phase. Additionally, our findings’ stronger alignment with CFIR 2.0 supports the importance of CFIR 2.0 updates, namely the development of the 1) *Outer Setting* domain to include *Partnerships & Connections*, *Local Conditions*, *Local Attitudes*; 2) *Inner Setting* and *Individuals* domains to understand *Teaming* processes; and 3) *Implementation Process* domain to include *Assessing Needs* (54). “*Rationale for the Intervention*” was a salient code that did not map onto CFIR 1.0 or 2.0. The issue seeming to undergird the distinction of this code from related CFIR constructs (e.g., *Need* in the *Individuals* domain) is that who should be the innovation recipients (those who attend OEND programs) and beneficiaries (those among whom naloxone is administered) was contested in our sample. Administrators differed on whether university-based programming should consider students when they are off-campus and members of the surrounding community, state, region, and nation. A key question that collaborators working to implement university-based OEND programs should consider is, “Whose needs matter to and fall under the responsibility of university administration, and is that purview malleable?”

### Building on Recent CFIR-Informed Research

Our findings build on our CFIR-informed qualitative focus groups conducted among purposively selected campus stakeholders (e.g., primarily students and some staff) on an urban university campus preparing to implement its OEND program (40). That work was limited to the experience of one university whose administration had already agreed to implement an OEND program, per the recommendations of a substance use committee that was part of an ongoing initiative to improve undergraduate mental health and substance use services. The current study extends our prior work by advancing understanding of OEND program implementation determinants across universities nationally, including key functions of leading substance use committees and determinants among universities whose administration has not already agreed to implement an OEND program. Further, our prior and current study address the lack of theoretically-informed implementation research on university-based OEND programs (46), and the limited application of implementation science theories and frameworks in similar settings (e.g., community-based OEND programs, university-based substance use programs) (47–52).

As a key example of how our current study extends our prior study, both underscore low perceived need for university-based OEND programs amidst competing student health priorities and perceived low student opioid misuse. To address that barrier, a recommendation from our prior study was to emphasize OEND programs’ applicability to communities surrounding the universities and students’ home communities (40). Our current study highlights additional considerations for this recommendation, especially when the university is not well integrated into or does not feel obligated toward those outside the campus. These findings indicate that internal substance use committees need to build a compelling case to convince university administration to implement an OEND program, and focusing on broad applicability is likely insufficient for administrators who consider their responsibility to be limited to students on campus. Such administrators may be more likely convinced by the increasing risk of unintentional, incidental opioid exposure among their students, relayed by administrators from universities that endured unexpected student opioid overdose deaths before implementing an OEND program.

### Emerging University-Based OEND Program Case Studies and Implementation Research

Emerging case studies of university-based OEND programs (45, 60, 61) and other AYA-focused OEND programs (62), as well as theoretically-informed (namely, CFIR) implementation research on a) community-based OEND/harm reduction programs (63–65) and b) university- and school-based programs that are likewise politically contentious (e.g. HIV prevention) (66–69) align with our findings. The key point of alignment is the importance of internal and external partners/collaborators conducting key implementation processes (e.g., *Planning*, *Engaging*, *Teaming*, *Assessing Needs*) to navigate implementation barriers within and outside of the campus, with student leadership and engagement emerging as a key adoption/implementation facilitator (61, 70, 71). A 2024 systematic review of university-based OEND programs corroborates the importance of collaborative implementation (46). Further, evidence of inter-university collaboration is emerging, pointing to a potential national community of practice (61, 71). Similar multi-stakeholder collaboration significantly increased community-level naloxone distribution through OEND program scale-up in the HEALing (Helping to End Addiction Long-term) Communities Study, a trial among 67 urban and rural communities across 4 states severely impacted by the opioid epidemic that is grounded in implementation science frameworks (72–75).

Universities across implementation phases can reference partner/collaborator roles identified in our findings and in this emerging literature as an initial model for ways in which existing or new, internal or external substance use coalitions can navigate the complexities of OEND program implementation. For example, building on the recovery-harm reduction trajectory identified in our findings, collaborators working to implement university-based OEND programming could make a compelling case to university administration and local law/policymakers that a dual recovery-harm reduction approach is vital to effectively reducing opioid overdose mortality (15–19, 76, 77), potentially overcoming the political controversy and stigma surrounding harm reduction programming that our participants described.

### Engaging University Administration

As part of this collaboration, our findings highlight the need to overcome university administration’s resistance to adopting/implementing OEND programs. Potential strategies include explaining: 1) the opioid misuse-suicidality correlation among college students (*Rationale for the Intervention*, emergent code in the *Innovation* domain) (78–80); 2) university counseling centers are strategically positioned to serve as implementation partners, as they typically receive institutional priority and funding (*Relational Connections*, *Inner Setting* domain) (81, 82); and 3) implementing OEND programs could help universities fulfill their institutional duty of care, as preventable student deaths create substantial reputational and legal risks (*Local Attitudes*, *Outer Setting* domain) (83, 84).

### Engaging Students in Need

Our findings highlight the need to overcome difficulties of identifying and *Engaging* (*Implementation Process* domain) students experiencing high opioid misuse or at risk of incidental exposure. As potential evidence-based *Design* (*Innovation* domain) strategies, universities have begun implementing in-person, virtual, and hybrid OEND programs and innovative outreach to engage hard-to-reach populations who might be at elevated risk of opioid overdose, within and beyond physical campus boundaries. This includes social media campaigns and naloxone distribution within known, non-stigmatizing, university infrastructure (e.g., automated external defibrillator boxes), which could extend to community infrastructure (e.g., harm reduction vending machines) (60, 61, 63, 71, 85–88).

### Limitations

Data were collected in 2020, and university administration perceptions of university-based OEND programs could have differed during the pandemic and since shifted considering recent work on the opioid overdose epidemic’s impact on AYAs (6, 27–32, 35, 37, 38). However, our findings showed awareness of opioid misuse/overdose among students was sometimes insufficient to move administration to implement an OEND program. Further, the university setting is still underutilized for OEND programs, suggesting our findings are relevant and actionable. A second limitation is that while we purposively selected a diverse sample of college health administrators, our findings might not be representative of all universities in the United States. Relatedly, our national sample was limited to the perspective of college health administrators and did not include other key stakeholders. A third limitation is that our enhancement of CFIR with the EPIS framework was broad, including only non-implementing and implementing universities and did not identify implementation determinant differences by each EPIS phase.

## Conclusions

Despite these limitations, this is one of the first studies to provide in-depth insights from a national United States sample of college health administrators into determinants of adopting and implementing university-based OEND programs and contributes to growing literature on applying CFIR among diverse university/school contexts. Building on key distinctions we identified between non-implementing and implementing universities, future research should establish OEND program implementation phase in a nationally representative sample of universities (e.g., per the EPIS framework), advance understanding of implementation determinants and strategies distinguishing each phase (89), and establish university-based OEND program core components and best practices (90, 91). In doing so, and building on our findings on the importance of internal/external collaborators, this future work should engage diverse internal and external stakeholder groups nationally (e.g., students, campus safety officers, local emergency services, university leadership; potential and current external collaborators).

## Supplementary Material

Supplementary Files

This is a list of supplementary files associated with this preprint. Click to download.

- AdditionalFile1.docx
- AdditionalFile2.docx
- AdditionalFile3.docx

## Figures and Tables

**Table 1:** Purposive selection of universities and college health administrators

Purposive Selection Criteria at the University and Administrator Level	Sample Description^a^
1) Broad geographical distribution, including regions and states that have been severely impacted by the opioid overdose epidemic	Broad representation across the United States, encompassing the West, Midwest, South, and Northeast regions, including states severely impacted by the opioid overdose epidemic (e.g., Massachusetts, New York, Ohio, North Carolina). See Fig. 1 for a visual representation.
2) Inclusion of both public and private universities, to account for potential differences	Public universities: n = 13 (68.4%) Private universities: n = 6 (31.6%)
3) Inclusion of both secular and faith-based universities, to account for potential differences	Secular universities: n = 17 (89.5%) Faith-based universities: n = 2 (10.5%)
4) Inclusion of a range of institutional classifications and research designations, to account for potential differences	**Institutional Classifications** (per the Carnegie Classification of Institutions of Higher Education (92))^b^ Mixed Undergraduate/Graduate-Doctorate Large: n = 8 (42.1%) Mixed Undergraduate/Graduate-Doctorate Medium: n = 4 (21.1%) Professions-focused Undergraduate/Graduate-Master’s Large/Medium: n = 4 (21.1%) Mixed Undergraduate/Graduate-Master’s Large/Medium: n = 1 (5.3%) Professions-focused Undergraduate/Graduate-Doctorate Large: n = 1 (5.3%) Professions-focused Undergraduate/Graduate-Doctorate Medium: n = 1 (5.3%) Total Fall 2020 Enrollment (i.e., more information on the size dimension of the above institutional classifications (93)) Mean: 25,332 enrolled students Median: 20,720 enrolled students Minimum: 3,302 enrolled students Maximum: 74,795 enrolled students **Research Designations** (per the Carnegie Classification of Institutions of Higher Education (92)) Research 1: Very High Research Spending and Doctorate Production: n = 11 (57.9%) Research 2: High Research Spending and Doctorate Production: n = 4 (21.1%) No research designation: n = 4 (21.1%)
5) Inclusion of college health administrators with relevant experience, expertise, and decision-making power regarding university-based OEND program implementation^c^	Student health promotion: n = 11 (55.0%) Student health services: n = 5 (25.0%) Student substance use services specifically: n = 3 (15.0%) Residence/Campus Life: n = 2 (10.0%) Professor in Health Sciences = 1 (5.0%)

a The sample descriptions of criteria 2–5 include the 19 universities, represented by 20 participants (2 participants represented the same university). Those sample descriptions do not include the statewide higher education substance use consortium.

b There are three dimensions in the Institutional Classification: Award Level Focus, Academic Program Mix, and Size. Please see the cited Carnegie Classification of Institutions of Higher Education resource for in-depth definitions of each dimension.

c Counts of college health administrators across roles total to 22, rather than 20 (and likewise percentages total to more than 100%), due to 1 participant having both a health services and Residence/Campus Life role and another participant having both a health promotion and professor role.

**Table 2:** Themes and subthemes mapped onto CFIR 2.0, including differences by implementation phase

THEME, SUBTHEMES, AND CFIR MAPPING	ILLUSTRATIVE QUOTES	KEY DIFFERENCES BY IMPLEMENTATION PHASE
**Theme 1: Justification that Compels Administration to Act: Reactive Orientation and University Purview**		
**Subtheme A: Reaction to Student Opioid Overdose and Mortality Events** *Related CFIR 2.0 domains and constructs* *Relative Priority* (*Inner Setting* domain) *High/Mid-Level Leaders (Individuals* domain*)* *Rationale for the Intervention* (emergent code in the *Innovation* domain)	“There were reports from a local harm reduction group that had been going around…trying to promote naloxone awareness in the community that there had been multiple deaths of [our] students off campus during the winter break due to drug overdoses…So, those were really the impetus that helped…the committee on substance safety and overdose prevention to be formed, and it led to really swift progress…Before that winter, [the administration] had said, ‘Resident advisors can be educated on opioid overdose, but we don’t wanna have naloxone on campus.’ To all of a sudden, ‘Okay, great. We’ll train the resident advisors. We can have naloxone on the 24-hour desks.”—**Implementing university**	**Implementing Universities** Among implementing universities, student opioid overdose deaths constituted a strong impetus to swiftly implement or more strongly prioritize an OEND program. **Non-Implementing Universities** In contrast, multiple participants from non-implementing universities explained that administration did not implement an OEND program despite the occurrence of student opioid overdoses due to a low perceived need for the program (e.g., low opioid misuse prevalence, low number of opioid overdoses, and lack of opioid overdose death among students, off-campus student overdoses being beyond university purview, greater relevance of OEND programs to the surrounding community) along with myriad other barriers (e.g., strong competing priorities such as student suicidality and deaths, limited campus resources, the need for internal partnerships to institutionalize an OEND program, liability concerns, and stigma-related optics concerns).
**Subtheme B: Student Opioid Misuse Data** *Related CFIR 2.0 domain and construct* *Assessing Needs* (*Implementation Process* domain)	“The key would be for us to do…a really good job with the justification…Why do we need to do this? And, again, that would have to be backed up by really good data…And I would pull in as many pieces of data as possible…Prior to even beginning to develop anything, I would have a…couple-hours sit down with all the interested folks across campus…I would engage as many of those folks as possible, ask them for data sources. We would probably reach out to…the paramedic response teams and the police…because they can be powerful allies…[and use local] ambulance statistics…Especially now because of the [COVID-related] budget concerns…I would use that data to position this kind of training and the resources it would take to do it…within the priority system of trainings.”— **Non-implementing university** Interviewer: “Have you heard anything of multiple substance use, possibly using other substances that are laced with opioids?” Participant: “Yes. Again, just hearing from our students that we work with regularly.” Interviewer: “Have you heard anything about fentanyl? Or, what have you heard about fentanyl use on campus?” Participant: “Um, yeah, definitely hear about it, but I don’t have like, quantitative numbers to back that up…So, what we see from our numbers is [opioid] use comparatively is lower from other substances. But again, for at-risk population, that’s a higher number…So again, if you’re looking at the general population, it’s a lower percentage. Um, sometimes if you look at just those numbers, it’s negligible, so it doesn’t really paint a picture.”— **Implementing university**	**Non-Implementing Universities** Participants from non-implementing universities underscored the importance of sufficient student opioid misuse prevalence data to justify implementing an OEND program. However, there were cases among non-implementing universities in which representative data or anecdotal knowledge of rising or ongoing student opioid misuse were insufficient to motivate administration to adopt an OEND program. **Implementing Universities** As a caveat to non-implementing universities’ emphasis on data, participants from implementing universities tended to underscore the limitations of available data (e.g., selection bias due to social desirability or opioid misuse-related dropout) and the need to identify and reach student subgroups experiencing high opioid misuse or at risk of incidental exposure (e.g., fentanyl lacing) even if opioid misuse among the general student populace was low (e.g., by disaggregating and triangulating data, including qualitative data and anecdotal knowledge of at-risk subgroups through their involvement in other campus substance use programs).
**Subtheme C: Relevant Opioid Overdoses and Opioid Misuse** *Related CFIR 2.0 domains and constructs* *Local Conditions* (*Outer Setting* domain) *Tension for Change* (*Inner Setting* domain)	“Our governor had done an opioid task force because of things in [the state] and we had been a hub for a while…so…the governor had invited our president actually to serve on it before he had become president because he had been in another position at the university and so he’s been very committed to [the OEND program], and he lives in the area and wants to help the community as a state institution. That’s part of our mandate, to be involved with the community.”— **Implementing university** “Most…opioid deaths in the county are in impoverished, uneducated, unconnected areas. And so, I wasn’t sure that, for [our] county, they should put their dollars on our college students, you know? But instead, with limited resources and limited funds, focus it in on those communities that were hurting the most.” — **Non-implementing university**	**Implementing Universities** Participants described stronger community obligation and integration among implementing universities, which led them to have a broader purview of responsibility to justify adoption. They underscored the relevance of community opioid misuse/overdose to student opioid misuse/overdose and the opportunity for students to administer naloxone among themselves and members of the surrounding community (whether on or off campus). **Non-Implementing Universities** Participants from non-implementing universities discussed high opioid misuse or overdose prevalence in the surrounding community, local and statewide opioid/substance use coalitions of which the university was a member, a desire to better integrate with the surrounding community, and the occurrence of off-campus student overdoses. However, in contrast to implementing universities, they did not discuss the relevance of community opioid misuse/overdose to potential student opioid misuse/overdose, did not acknowledge the possibility of students administering naloxone in the surrounding community, or understood off-campus applicability, even among students, to be beyond university responsibility.
**Theme 2: External and Internal Partnerships are Needed to Support Adoption and Implementation**		
**Subtheme A: External Partnerships and Connections** *Related CFIR 2.0 domains and constructs* *Partnerships & Connections* (*Outer Setting* domain) *Planning, Engaging, Teaming* (three constructs in the *Implementation Process* domain)	“We’re just starting the conversation. So, I understand that there is [a local opioid overdose education and naloxone distribution] program…and a number of our students, I understand have been trained by [the program]…in the use of Narcan and also have it available to them…but I’m, again, I’m just learning…So, I’d really love to be able to partner with it so that it doesn’t feel like it’s outside of our system and not integrated into the response team that we have in place on campus.”— **Implementing university**	**Implementing Universities** Participants illustrated several critical reasons why external partners/collaborators would be critical to engage in efforts to adopt and implement OEND programs. Participants, primarily among implementing universities, perceived the following as key functions of these collaborations: **1)** overcoming administration’s active pushback or passivity; **2)** allowing students to access an OEND program elsewhere during the university’s Exploration or Preparation, which could lead to a sustained external partnership; **3)** strategically incorporating naloxone training and/or kits that are readily accessible in the surrounding community; **4)** overcoming funding/resource constraints; **5)** promoting sustainability; **6)** building academic-community relationships; **7)** staying informed on and responding to opioid misuse/overdoses in the surrounding community and among students; **8)** lowering barriers for students who use illicit drugs and do not wish to identify themselves in a university setting. **Non-Implementing Universities** This subtheme was primarily discussed by participants from implementing universities, but some participants from non-implementing universities described the importance of external partners/collaborators for **1)** overcoming administration’s active pushback or passivity; **2)** allowing students to access an OEND program elsewhere during the university’s Exploration; **4)** overcoming funding/resource constraints; **7)** staying informed on and responding to opioid misuse/overdoses in the surrounding community and among students.
**Subtheme B: Internal Partnerships and Connections** *Related CFIR 2.0 domains and constructs* *Relational Connections* (*Inner Setting* domain) *Planning, Engaging, Teaming* (three constructs in the *Implementation Process* domain)	“The barriers are…less about resources and more about sort of breaking through some of the misconceptions or misperceptions about like why would we need to do this? … You know, having enough will within the AOD, health education, student services to [move] more in that direction. And there’s will in pockets, but there isn’t enough will to make it…a part of the system…Do your relationship building throughout the implementation of your plan, from sort of planning all the way through to evaluation.”— **Non-implementing university** “The first meeting was really to…understand what other people knew. I’m relatively new and a bunch of other people who are stakeholders with me…are also new. And so, we, I needed to understand the history. I had understood that…this conversation had been visited once before several years ago and…I didn’t understand what happened…So I wanted to kind of interrogate that a little more and to understand what other people knew about this issue and how they felt about it, and…whether they thought it was feasible or not. And…get a sense of the roadmap, like where the barriers might be…Now that I have a sense of that and what people think would be practical and a way forward, I think that…it would be helpful to bring on some other people who…feel really passionate about it.” – **Implementing university**	**Non-Implementing Universities** Participants from non-implementing universities emphasized the need for internal partners to gather data and share perspectives to establish a need for an OEND program that would compel administration to adopt it. **Implementing Universities** Those from implementing universities described internal partners (namely, substance use committees) facilitating OEND program planning and implementation (e.g., discussing implementation determinants and strategies) and sustainability (e.g., championing the program, implementing new awareness and engagement strategies, creating social work student internships to support OEND program implementation, conducting program evaluation).
**Theme 3: Navigating External and Internal Culture**		
**Subtheme A: Navigating External Politics and Culture** *Related CFIR 2.0 domain and construct* *Local Attitudes* (*Outer Setting* domain)	“I really do feel it’s an institutional reluctance to take on this issue. It’s a political risk management issue, frankly…Cost is not so much an issue, but more about institutional reputation, and publicity, things like that.” — **Non-implementing university** “I think that for naloxone responding…it’s really, really important to think about the cultural differences that we have and meet that community…where they’re at. With understanding substance abuse prevention, what role the community has in addressing that substance abuse prevention. And not taking it too far that we alienate people, but moving them away from their stigma, but not moving them so far that they bounce back into that stigma.” – **Implementing university (statewide substance use consortium)** “The White savior problem that we ran into. Here we were, this agency funded by the state government, who is deciding…that we were going to give specific resources [to two historically black colleges and universities]. We thought we were doing a good thing…they didn’t have many resources…[There was] the targeting feeling of, “Okay, you’re assuming that this is a problem only in the Black community.” Which is when we had to then go back to the data and say, “Your data is telling us this…Opioid prevention is just as wrought with politics as the alcohol or tobacco or cannabis landscape, it’s just different than those other three.” – **Implementing university (statewide substance use consortium)**	**Non-Implementing Universities** Participants from non-implementing universities explained that political risk management concerns (e.g., optics related to regional/state culture, state legislatures/representatives, funders) could prevent adoption, even when administration is aware of ongoing student opioid misuse. **Implementing Universities** Among implementing universities, participants indicated that while political risk management would be or had been an important factor to address, it was feasible to handle (e.g., by framing OEND programs as widely applicable public health prevention rather than incrimination of an on-campus problem; by gradually increasing campus and community visibility of the OEND program).
**Subtheme B: Navigating Internal Culture** *Related CFIR 2.0 domain and constructs* *Culture* and *Available Resources* (*Inner Setting* domain)	“Obviously if we had…a collegiate recovery program, I think that would help too. So right now, one of my major focuses is starting that effort. And it’s very, very small right now. I’m just kind of gathering information for educational opportunities…I need to educate the campus on what is a recovery program. What does it mean to be a recovery ally? So, I think if we had had that in place that the whole education of heroin and opioids, and then the subsequent naloxone training and education…would have been a lot easier, so. — **Implementing university** “Within the communities that use these substances [e.g., cocaine and fentanyl], I would say that we have a fairly deeply ingrained drug culture in those groups. There’s a sense of mistrust of the university and…some secrecy around sharing, being upfront. I would say, though, that the community was really impacted by – three of the deaths were actually members of the Greek community [sororities/fraternities]. So, I think that the students, in responding to that, it really, I think changed some of the sense of – for our students, there’s a sense that cocaine use is very safe. There wasn’t a sense that it was something that was a high-risk activity. It was very normalized. So, we’ve been able to have some good conversations with them about that.” – **Implementing university** “I think [implementing an OEND program] is [a] very low [priority]. Just being completely transparent. I think, honestly, even alcohol, for as big of an issue as it is here, again, given sort of the pervasive attitudes with faculty, staff, students, alumni and parents, it’s one of those things that they know we need to do. They know it’s our biggest substance abuse issue on campus. But it’s also a ‘wink, wink’. We know we need to do this, but we also know there are things that go on. So, I think that because of the cavalier attitude around alcohol, I think it diminishes sort of the substance abuse issue as a whole here…I think if we were to ever find ourselves in a position where we have the student buy-in first around this conversation related to recovery, I think that the conversation around naloxone and expanding training to other substances and supports would really support that conversation…I think there could be synergy. And I raise that only because it’s the only kind of connected conversation that we have had here in recent years that just has not gotten any legs. So, for me, on this particular campus, if we were to get legs around one of those conversations, I think the other might follow.” – **Non-implementing university** “I do think that having [naloxone-related] resources on campus, on some level, I think we’ll probably go that direction at some point…I think we could be doing more in that sense, in terms of integrating that option, the naloxone is more in [line] with our stated goals of supporting an environment of protection [for] students.” – **Non-implementing university**	**Implementing Universities** Participants from implementing universities illustrated a trajectory in which their institutions prioritized recovery programming for years before prioritizing harm reduction programming. **Non-implementing universities** This recovery-harm reduction trajectory likewise emerged among two non-implementing universities that had instituted substantial recovery programming and were beginning to institute harm reduction programming, with participants describing the laborious, time-consuming processes required. However, other non-implementing universities were not at the point of prioritizing recovery or other substance use programming, which participants perceived as a substantial barrier to adopting an OEND program.

## Data Availability

The datasets generated and/or analyzed during the current study are not publicly available to protect participant confidentiality (e.g., publishing interview transcripts would violate participant confidentiality, regardless of redaction), but additional illustrative quotes could be made available from the corresponding author on reasonable request.

## Abbreviations

ACHA: American College Health Association
ACHA-NCHA: American College Health Association-National College Health Assessment
AYAs: Adolescents and young adults
CFIR: Consolidated Framework for Implementation Research
EPIS Framework: Exploration, Preparation, Implementation, Sustainment Framework
HEALing Communities Study: Helping to End Addiction Long-term Communities Study
HIV: Human immunodeficiency virus
NASPA: Student Affairs Administrators in Higher Education
OEND program: Opioid education and naloxone distribution program
PWUD: People who use drugs
